# Mismatch Negativity Affects Muscle Fatigue during Repeated Contraction Trials of Different Durations

**DOI:** 10.3389/fphys.2016.00013

**Published:** 2016-02-01

**Authors:** Aleksander A. Aleksandrov, Veronika M. Knyazeva, Ludmila N. Stankevich, Elena S. Dmitrieva, Anna N. Shestakova

**Affiliations:** ^1^Department of Higher Nervous Activity and Psychophysiology, Saint Petersburg State UniversitySaint Petersburg, Russia; ^2^Centre for Cognition and Decision Making, National Research University Higher School of EconomicsMoscow, Russia

**Keywords:** central muscle fatigue, attention, event-related potentials, mismatch negativity, cognitive load

## Abstract

We examined the effect of involuntary attention switching (related to mismatch negativity generation in the oddball paradigm) on fatigue development during trials of different durations. The experiment consisted of two trials, long (40 min) and short (15 min), and two experimental conditions in each trial: the simple reaction task (deviants-only paradigm) and the stimuli recognition task (oddball paradigm). In each condition, a participant responded to each target acoustic stimulus by squeezing a handgrip dynamometer. We found the significantly lower rates of fatigue development in the short-trial deviants-only paradigm compared to the long trial. The short- and the long-trial oddball paradigms differed significantly from both the short- and the long-trial deviants-only paradigms. The results demonstrated that the fatigue developed differently depending on the expected trial duration. The involuntary activation of attention broke this subconscious regulative mechanism leading to increase of the compression force during the long trial and its decrease during the short.

## Introduction

Muscle fatigue is defined as an exercise-induced reduction in the force-generating capacity of the neuromuscular system (Birgland-Ritche and Woods, 1984). Changes occurring at the spinal and supraspinal levels have a strong influence on the development of fatigue. This process is known as central fatigue, that is, the lack of an optimal efferentation from the central nervous system (Gandevia, 2001).

It was shown that central muscle fatigue and cognitive processes are connected. Firstly, the development of muscle fatigue influences some cognitive processes (Lorist et al., 2002; Davranche and Pichon, 2005; Zijdewind et al., 2006). For example, in the dual-task paradigm the simultaneous performing of sustained submaximal contraction (fatiguing task) and auditory choice reaction task (cognitive task) resulted in a decline of accuracy and speed of cognitive work (Lorist et al., 2002; Zijdewind et al., 2006). Authors have claimed that limited resources of voluntary attention are distracted by an increasing perception of fatigue. On the other hand, muscle fatigue may impair the involuntary stages of auditory information processing. The reduction of mismatch negativity (MMN) amplitude in frontal and central areas occurs during the sustained submaximal contraction (Evstigneeva et al., 2010).

Secondly, development of fatigue and perceived exertion could be also reduced by different cognitive factors, such as motivation (Tanaka et al., 2013; Blanchfield et al., 2014), listening to music (Barwood et al., 2009; Lim et al., 2009), and hypnosis (Morgan, 1973; Morgan et al., 1976). The recovery after fatigue may be accelerated by physical or mental activities during the pauses (Mathiassen et al., 2008). It was also shown that diversity and variation of muscle load alleviated fatigue caused by sustained or monotonous repetitive work (Mathiassen, 2006). The performance of a cognitive task (auditory choice reaction task) simultaneously with a fatiguing motor task (sustained submaximal contraction) results in improved performance of the motor task in comparison with the passive listening task (Evstigneeva et al., 2012). We can assume that at least some of these processes are associated with attention.

Attention is a very important and complicated cognitive function with a variety of different aspects. Basic neurophysiological mechanisms of attention are dichotomized into voluntary and involuntary processes. Involuntary attention refers to the phenomenon of switching attention to a new object (Näätänen, 1986). One of the best-studied neurophysiological mechanisms of the involuntary attention system, recorded in human-evoked brain potentials, is mismatch negativity (Näätänen et al., 2011). MMN occurs whenever an acoustic signal differs in some measure from the standard stimuli presented to the subject. This auditory change forms a new input to the sensory memory that does not correspond with the previous inputs formed by the standard stimuli and triggers a neuronal generator, resulting in the MMN wave (Näätänen et al., 1989). The MMN wave is some kind of automatic sensory information–processing mechanism at a pre-attentive stage, which may play an important role in the involuntary attraction of attention (Näätänen and Gaillard, 1983; Tiitinen et al., 1994). Data from our laboratory show that the neurophysiological mechanisms of MMN generation may have an activating effect (Aleksandrov et al., 2003, 2005); conversely, dampened MMN was associated with increased reaction time and the number of errors in the acoustic signal recognition task.

Therefore, one may suppose that the fatigue development variations in some paradigms are associated with the activation of attention system. However, there are no clear data about the influence of involuntary attention on the development of muscle fatigue. We assume that the involuntary attention related to the generation of MMN can reduce the development of fatigue. Therefore, the aim of the present study was to examine the effect of involuntary attention related to MMN generation on fatigue development during trials of different durations.

## Methods

### Participants

Eighteen healthy adults (7 men and 11 women), ranging in age from 20 to 26 years old, took part in the long trial (LT) of the experiment and in the short trial (ST) there were 11 adults (2 men and 9 women) ranging in age from 23 to 37 years old, who did not participate in the LT. During the experiment, electroencephalograms (EEGs) were recorded from the first eight participants of the LT. All subjects had normal or corrected-to-normal vision and intact hearing. All subjects were right-handed and used the dominant hand when performing the motor task. One subject was excluded from the EEG analysis because of numerous artifacts. All subjects gave written informed consent prior to the study. All experimental procedures were approved by the local ethics committee (Saint Petersburg State University, Russia) and conducted according to the Declaration of Helsinki.

### Procedure

#### Protocol

**Figure 1:**
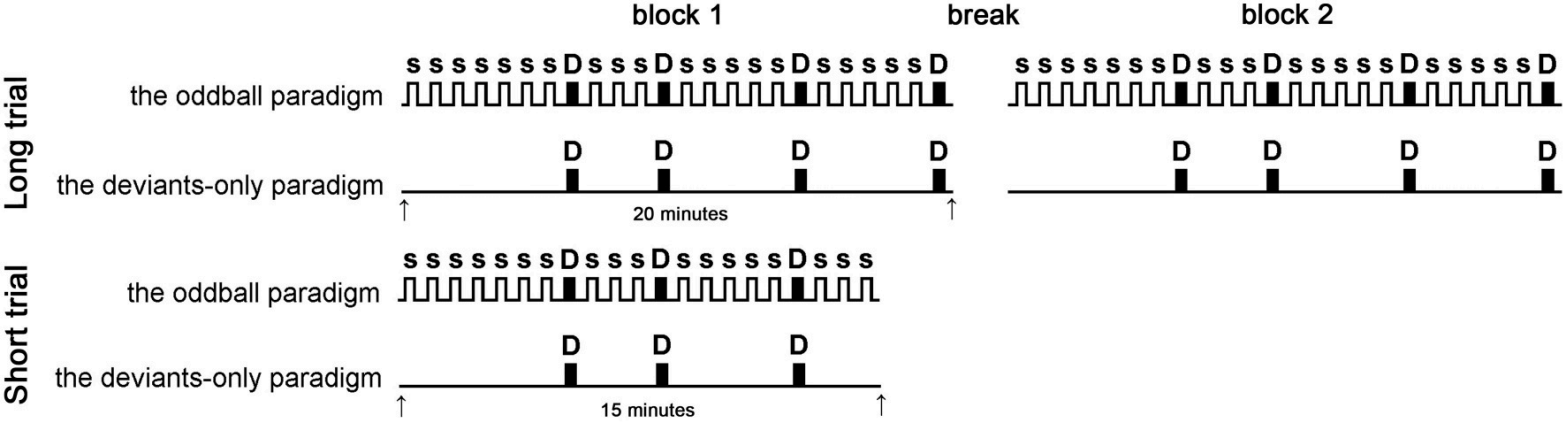
**Experimental design**. The experiment consisted of four conditions: long trial (LT) deviants-only, LT oddball, short trial (ST) deviants-only, and ST oddball paradigms. LTs consisted of two identical 20-min blocks, separated by a break, while STs consisted of single 15-min blocks. The figure shows the stimuli sequence: S = nontarget stimulus (1000 Hz), D (shaded) = target stimulus (1200 Hz). Target stimulus was presented at the same times in each condition. The subject was instructed to compress the hand dynamometer in response to target stimulus.

The experiment consisted of two trials (Figure 1). The LT consisted of two identical 20-min blocks, separated by a 3-min break; the ST consisted of one 15-min block. Participants were informed about the task duration, but were not informed about the time remaining within the trial.

Each block was a sequence of acoustic stimuli presented in two paradigms: the deviants-only paradigm and the oddball paradigm. In each condition, the participant was instructed to respond to the target acoustic stimulus by squeezing the working part of the handgrip dynamometer (KRG-4 T10, Nobel Elektronik, Karlskoga, Sweden). The deviants-only paradigm (control) was the simple reaction task where each stimulus was the target. The oddball paradigm consisted of two types of auditory stimuli one much less frequent than the other. In this paradigm the participant performed the stimuli recognition task responding on the rare target stimuli and ignoring the frequent nontarget. The oddball paradigm led to the generation of the MMN (Näätänen et al., 2004); this reflects the activation of the pre-attentive mechanism that detected changes in the acoustic environment (Näätänen et al., 2011). As was mentioned in the Introduction, this process may have an activating effect. Therefore, there were four conditions: the LT deviants-only, the LT oddball, the ST deviants-only, and the ST oddball paradigms. The number of target stimuli was equal in each condition. The interval between the target stimuli ranged from 2.5 to 7.55 s. The conditions were each conducted on a separate day and were counterbalanced between subjects.

The participant sat behind the experimental table with the right forearm on the armrest and the working part of the handgrip dynamometer in the hand. The dynamometer was connected with the computer that provided the force feedback. Lines on the computer screen showed the target level and the real-time force of compression. The target level was the value of the maximal voluntary contraction (MVC) measured before the start of the block. The participants were instructed to compress the dynamometer with a short quick squeeze and with maximal effort until the line reached the target level, or less if they were fatigued.

MVC was recorded at the beginning and at the end of each block and measured as the maximum sustained dynamometer compression, averaged over 5 s. The force of the dynamometer compression was measured in units (one unit = 653 Newton). The rating of perceived exertion (RPE) of the working hand muscles was measured before the MVC according to Borg's scale: the 10-point scale varies from 1 (do not feel muscle fatigue) to 10 (intolerable muscle fatigue; Borg, 1998). The decrease in amplitudes of short quick squeeze compressions, MVC and RPE values before/after blocks was used as fatigue indicators.

The same demand for motor performance in all parts of the experiment was achieved by using a similar scheme for the stimulation: the occurrence of target stimulus was fixed in all three paradigms (Figure 1); that means that target stimulus was presented at the same time (e.g., at the 6th, 9th, and 14th s from the start of the experiment) in each paradigm, regardless of how many times nontarget stimulus (if at all) occurred between them.

#### Stimuli

The two types of stimuli, nontarget (1000 Hz tone) and target (1200 Hz tone), were presented binaurally via speakers (duration: 50 ms, including 5 ms rise/fall interval; intensity: 60 dB SPL). For the creation and presentation of stimuli, Psytask v. 1.41.2 software (Mitsar Co. Ltd., St. Petersburg, Russian Federation) was used.

The deviants-only paradigm consisted of two blocks with 225 target stimuli for the LT of the experiment and one block with 200 target stimuli for the ST. The oddball paradigm consisted of two blocks with 1350 stimuli, non-target (standard) – 1125 (83%) and target (deviant) – 225 (17%) in the LT, and one block with1180 stimuli in the ST, non-target (standard) – 980 (83%), and target (deviant) – 200 (17%); the inter-tone interval was 800 ms. The deviant stimuli probability value was used to reduce the contribution of refractoriness in the MMN (differences in the refractoriness of neuron populations responding to different stimuli; Opitz et al., 2005). We chose the 17% probability of deviant stimuli because it appeared to be optimal and agreed with the results of previous studies (Evstigneeva and Aleksandrov, 2009).

#### Data analysis

Three parameters of fatigue development, the dynamometer compression force in response to target stimulus, the value of MVC, and the rating of perceived exertion were analyzed. The graph of compression force for each iteration of target stimulus was recorded by the use of Force Feedback v. 2.0 (National Instruments Corporation, TX, Austin, USA) and then converted to Spike 2 v. 6.05 (Cambridge Electronic Design, Cambridge, UK). The maximal amplitude of every graph was automatically measured. These amplitudes were averaged in a series of 10 consecutive values and calculated as the percentage of the mean of the first five values. Thus, 45 averaged blocks were obtained for each condition in the LT of the experiment and 20 averaged blocks for each condition in the ST. The MVC value was recorded at the beginning and end of the block and was measured as the maximum compression of the dynamometer, averaged over 5 s. The level of RPE was measured according to Borg's scale before and after the block. MVC and RPE were calculated as the means, and after that, as the percentage of the first value.

#### Electrophysiological data recording and analysis

The EEG recording was performed by using 11 silver-silver chloride electrodes placed at the F3, Fz, F4, C3, Cz, C4, P3, Pz, and P4 positions, according to the international 10–20 system (Jasper, 1957), and both mastoids (M1, M2). The reference electrode was on the tip of the nose, and the ground on the forehead. The electrooculogram was recorded with the electrode placed at the lateral canthus of the left eye. The electrode resistance did not exceed 5 kΩ. The EEG was recorded with a Mitsar-EEG-05/70-201 21-channel digital EEG amplifier (Mitsar Co. Ltd., St. Petersburg, Russian Federation; bandwidth 0.16–70 Hz, sampling frequency 500 Hz) and the WinEEG v. 2.4 software (Mitsar Co. Ltd., St. Petersburg, Russian Federation).

The EEG signal was filtered in a 0.5–30 Hz band-pass filter. The duration of the analyzed EEG epoch was 600 ms, including a 50 ms interval before the stimuli, which was subsequently used for the correction of the baseline. Epochs where the signal amplitude exceeded ±100 μV in any channel were excluded from averaging. The amplitudes of the auditory event-related potential (ERP) components were measured as the mean amplitude (baseline to peak) in the latency range of the ERP optimal intensity. N1 was measured in the 88–138 ms range for all paradigms. For the oddball paradigm, P2 was measured in the 152–178 ms range, N2b in the 182–242 ms range, and P3 in the 280–330 ms range. For the deviants-only paradigm, P2 was measured in the 178–208 ms range, and P3 in the 312–362 ms range.

### Statistics

Data were analyzed using IBM SPSS Statistic v. 21 (IBM Corporation, New York, USA). All data were normally distributed and that was confirmed by the Kolmogorov-Smirnov test. The force of compression was compared between conditions separately within LTs and STs using two-way repeated-measures analysis of variance (ANOVA) with average block (45 levels for the LT; 20 levels for the ST) and condition (2 levels) factors. The Greenhouse–Geisser correction was made where appropriate. When the main analysis indicated a significant effect of a factor or an interaction between factors, a *post-hoc* pairwise comparisons test with Bonferroni adjustment for multiple comparisons was made within conditions and the paired-samples *t*-test with correction for multiple comparisons according to false discovery rate criteria (FDR) was made between conditions. The MVC and the RPE for the LT were analyzed by two-way ANOVA with measurement number (4 levels) and condition (2 levels) as factors. The MVC and the RPE for the ST were analyzed using the paired-samples *t*-test. LTs and STs were compared using the independent-samples *t*-test. For the force of compression, the first 20 measures of the LT were compared with 20 measures of the ST, using repeated-measures ANOVA and independent-samples *t*-test with correction for multiple comparisons according to FDR criteria. For the ERPs' amplitudes in oddball paradigms, the factors in the ANOVA were lead (11 levels), stimulus (2 levels), and block (2 levels). For the ERPs' amplitudes in the deviants-only paradigm, the factors were lead (11 levels) and block (2 levels).

## Results

**Figure 2:**
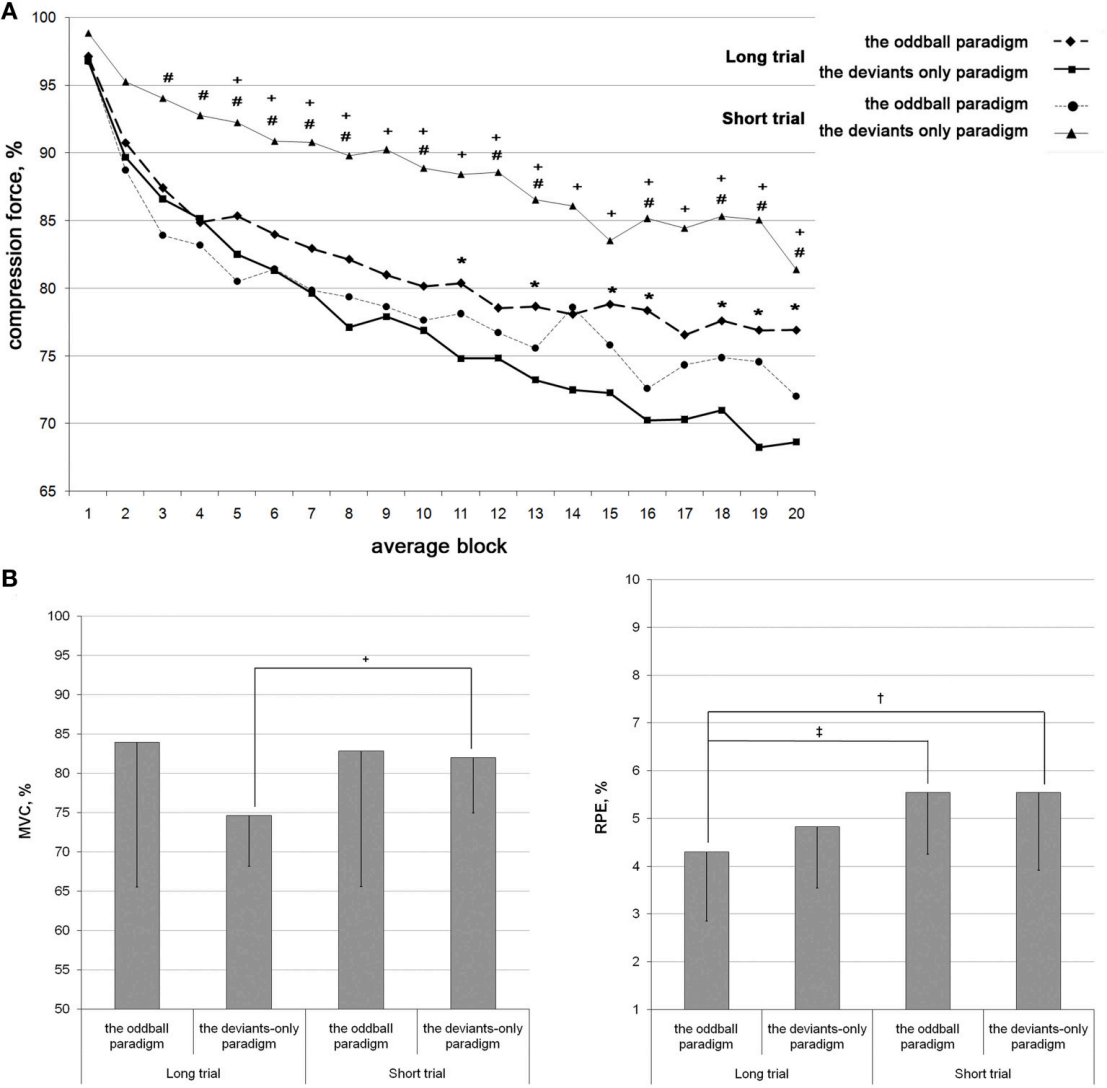
**Fatigue development during the task performance in four conditions with known duration: the LT deviants-only, the LT oddball, the ST deviants-only, and the ST oddball paradigms. (A):** The force of short dynamometer compression in response to the target stimulus averaged in series of 10 consecutive values (averaged blocks) and calculated as the percentage of the mean of the first five values. The first 20 blocks of LT and 20 blocks of ST are shown. **(B)**: The MVC and the RPE values calculated as the means, and after that, as the percentage of the first value. The bars indicated the standard deviations. ^*^p ≤ 0.05 between the LT oddball and the LT deviants-only paradigms; ^#^*p* ≤ 0.05 between the ST oddball and the ST deviants-only paradigms; ^+^*p* ≤ 0.05 between the ST deviants-only and the LT deviants-only paradigms; ^‡^*p* ≤ 0.05 between the LT oddball paradigm and the ST oddball paradigms; ^†^*p* ≤ 0.05 between the LT oddball and the ST deviants-only paradigms.

Since the fatiguing task was identical in different conditions, the difference between conditions was only in the activation of the pre-attentive MMN mechanism. Figure 2 illustrates the changes of dynamometer compression force, MVCs, and the rating of perceived exertion during performance of the fatiguing task in two conditions of LTs and STs. The means and standard deviations (SD) of the dynamometer compression force are shown in the Table 1.

**Table 1:** **The compression force means and standard deviations**.

**Average block no**.	**The ST deviants-only paradigm**		**The ST oddball paradigm**		**The LT deviants-only paradigm**		**The LT oddball paradigm**	
	***Mean***	***SD***	***Mean***	***SD***	***Mean***	***SD***	***Mean***	***SD***
1	98.84	3.05	96.76	3.85	96.80	3.87	97.11	3.62
2	95.23	9.67	88.71	8.10	89.67	8.94	90.75	9.07
3	94.04	13.22	83.91	8.98	86.59	8.59	87.40	11.39
4	92.76	11.94	83.17	7.59	85.15	8.71	84.85	11.67
5	92.23	15.57	80.50	7.47	82.50	8.42	85.35	13.07
6	90.87	14.00	81.41	8.50	81.31	8.24	83.99	12.73
7	90.79	16.37	79.84	7.21	79.63	9.35	82.94	13.89
8	89.79	16.30	79.35	10.09	77.11	8.80	82.13	11.66
9	90.23	17.40	78.62	9.87	77.90	8.21	80.98	12.20
10	88.87	16.54	77.64	9.28	76.88	10.06	80.15	12.07
11	88.42	18.23	78.11	9.95	74.81	10.05	80.37	13.44
12	88.56	17.72	76.72	9.84	74.83	10.47	78.53	12.60
13	86.52	16.33	75.58	11.70	73.23	11.15	78.65	12.70
14	86.08	16.69	78.60	7.75	72.49	10.58	78.07	12.64
15	83.52	16.43	75.81	9.97	72.27	10.79	78.83	13.02
16	85.15	17.20	72.60	11.81	70.25	12.62	78.37	12.30
17	84.44	18.31	74.33	10.73	70.31	12.22	76.55	14.17
18	85.33	18.10	74.87	8.64	70.98	12.77	77.60	14.75
19	85.04	16.30	74.55	8.21	68.25	13.89	76.90	14.50
20	81.37	18.28	72.02	12.10	68.64	13.57	76.92	12.66
21					68.60	14.82	76.57	12.40
22					67.63	14.52	76.85	13.82
23					78.22	11.68	89.42	13.43
24					72.81	13.42	82.54	13.58
25					71.18	14.08	79.27	12.97
26					69.61	13.35	77.74	13.08
27					67.60	13.90	76.04	13.13
28					66.70	13.16	74.33	14.53
29					64.96	13.78	74.98	14.40
30					64.91	14.32	73.18	15.66
31					65.33	13.75	72.27	14.01
32					64.48	15.64	73.60	14.69
33					65.63	16.01	71.17	14.22
34					63.25	15.12	71.17	12.60
35					61.62	15.16	70.93	13.42
36					61.59	14.54	70.94	14.08
37					62.50	13.71	70.72	12.86
38					61.60	15.03	71.05	12.92
39					60.16	14.11	71.67	13.73
40					60.25	14.65	72.11	15.01
41					61.85	14.14	71.74	14.02
42					62.63	12.17	71.77	13.84
43					61.37	13.17	71.38	12.89
44					61.00	12.99	70.88	12.10
45					61.08	12.06	73.50	11.60

*The compression force amplitudes were averaged in a series of 10 consecutive values and calculated as the percentage of the mean of the first five values. 45 averaged blocks were obtained for each condition in the LT (22 for the 1th block and 23 for the 2nd) and 20 averaged blocks for each condition in the ST*.

### Compression force

#### The LT

There was a progressive decrease in the force of compression: the two-way ANOVA revealed a significant effect for the average block [*F*_(3.625)_ = 37.777; *p* < 0.001]. During the development of fatigue, the compression force was larger in both blocks of the *oddball* [*F*_(1)_ = 7.148; *p* = 0.019].

#### The ST

There was a progressive decrease in the force of compression: the two-way ANOVA revealed a significant effect for the average block [*F*_(3.157)_ = 20.246; *p* < 0.001]. During the development of fatigue, the compression force was larger in the *deviants-only* paradigm [*F*_(1)_ = 6.580; *p* = 0.028].

#### The LT vs. the ST

There was a significant difference between the LT and the ST deviants-only paradigms: during the ST, the force of compression was significantly larger than during the LT [*F*_(1)_ = 7.990; *p* = 0.009]. No differences were found between the LT and the ST oddball paradigms, the LT oddball and the ST deviants-only paradigms, and the LT deviants-only and the ST oddball paradigms. Therefore, despite being asked to perform the same maximal exertion in absolutely identical motor tasks, subjects displayed significantly lower rates of fatigue development in the ST deviants-only paradigm than the LT [*F*_(1)_ = 7.990; *p* = 0.009]. The ST and the LT oddball paradigms took an intermediate position, differing significantly from the ST and the LT deviants-only paradigms, respectively [*F*_(1)_ = 6.580; *p* = 0.028; *F*_(1)_ = 7.148; *p* = 0.019].

### MVC

#### The LT

In the MVC data there was a significant effect of measurement number [*F*_(3)_ = 55.773; *p* < 0.001]. *Post-hoc* analysis revealed a significant difference between the 1st and 2nd, 3rd, and 4th measures (*p* < 0.001), 2nd and 3rd measures (*p* = 0.031), 2nd and 4th measures (*p* = 0.002), and 3rd and 4th measures (*p* < 0.001). Therefore, MVC data revealed the development of fatigue: the MVC values decreased, rising after the period of rest and decreasing after each period of work. MVC values for the 3rd measure were significantly larger in the oddball paradigm than the deviants-only paradigm (*t* = 2.548, *p* = 0.022).

#### The ST

MVC values decreased after the block: there was a significant difference between the 1st and 2nd measures in the oddball (*t* = 3.312, *p* = 0.008) and deviants-only (*t* = 8.463, *p* < 0.001) paradigms. No significant differences were found between the MVC values in the paradigms.

#### The LT vs. the ST

The MVC values measured after the first block were significantly larger for the ST than for the LT in the deviants-only paradigm (*t* = 2.802, *p* = 0.010). There was no significant difference between the ST and LT oddball paradigms and between the ST oddball and LT deviants-only paradigms. Therefore, the MVC data, as well as compression force data, showed a lower rate of fatigue development in the ST than the LT (*t* = 2.802, *p* = 0.010), but only in the deviants-only paradigm.

### RPE

#### The LT

The RPE data showed significant effects for the measurement number [*F*_(3)_ = 107.770; *P* < 0.001] and paradigm [*F*_(1)_ = 4.754; *P* = 0.045]. RPE values increased, decreasing after the period of rest and rising after each period of work: *post-hoc* analysis revealed a significant difference between the 1st and 2nd, 3rd, and 4th measures (*p* < 0.001), 2nd and 3rd measures (*P* < 0.001), 2nd and 4th measures (*p* = 0.013), and 3rd and 4th measures (*p* < 0.001). RPE values for the 3rd measure were smaller in the oddball paradigm than the deviants-only paradigm (*t* = −2.853, *p* = 0.011).

#### The ST

RPE values increased after the block: there was a significant difference between the 1st and 2nd measures in the oddball (*t* = −11.656, *p* < 0.001) and deviants-only (*t* = −9.221, *p* < 0.001) paradigms. There was no significant difference between the paradigms regarding RPE values.

#### The LT vs. the ST

In the LT oddball paradigm, the RPE values measured after the first block were lower than those in the ST oddball (*t* = −2.326, *p* = 0.028) and ST deviants-only paradigms (*t* = −2.124, *p* = 0.043). Therefore, RPE values were smaller in the first block of the LT but matched those of the ST in the second block; at the ends of the STs and LTs, RPE values were similar.

### Event-related potentials

**Figure 3:**
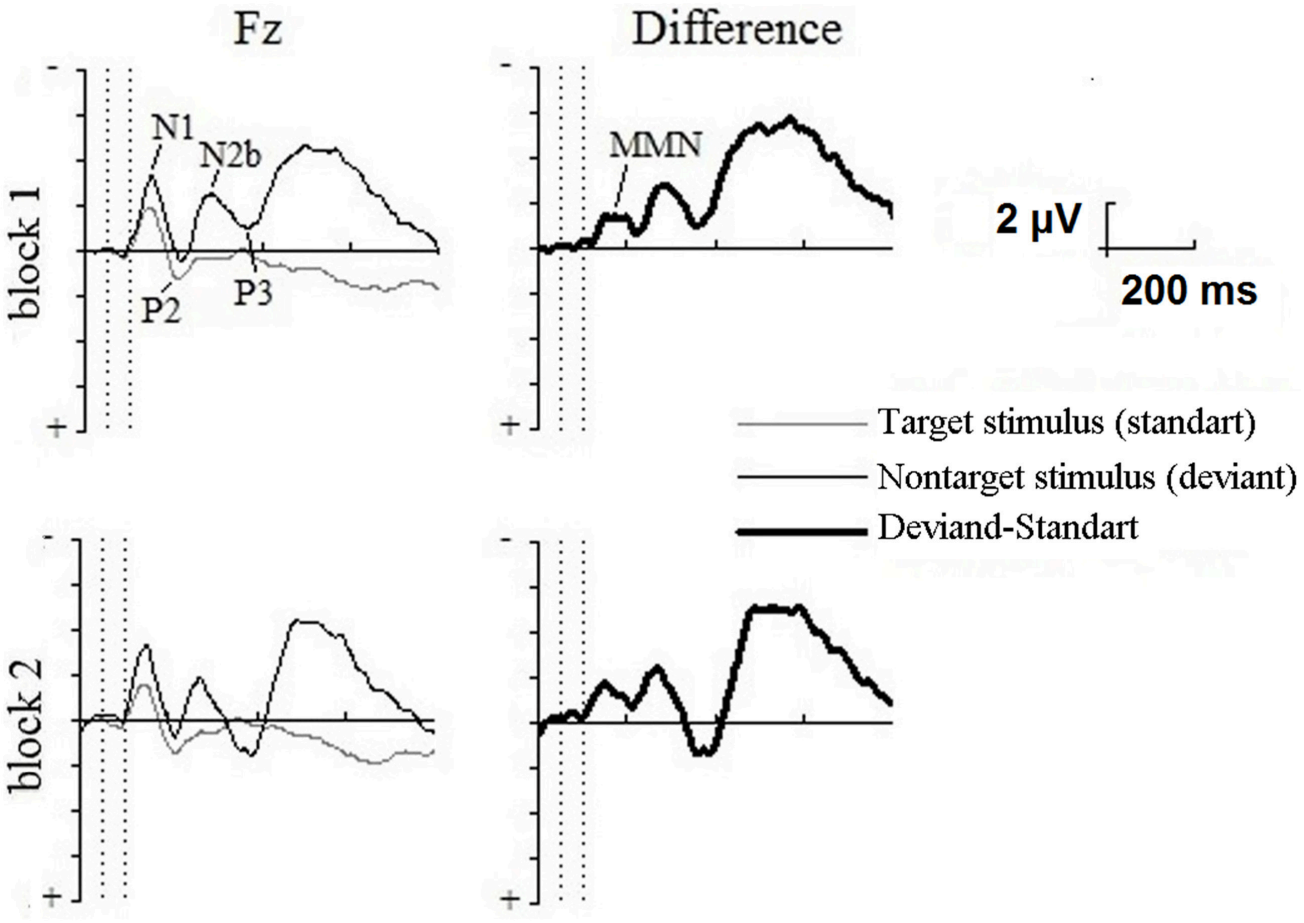
**Grand average across participants frontal (Fz) event-related potentials in the LT oddball paradigm for the first (above) and second (below) blocks to nontarget stimulus (standards), target stimulus (deviants), and the difference waves data, that were obtained by subtracting the standard stimulus ERP from that of the deviant stimulus**. Vertical dotted lines show the beginning and end of the stimulus. Evoked potential components are identified on the first block. The data were referenced to the nose electrode.

The following components of auditory ERPs were identified in the oddball paradigm: N1 and P2 in response to nontarget stimulus (standard); N1, P2, N2b, and P3 in response to target stimulus (deviant). The MMN was obtained by subtracting the standard stimulus ERP from that of the deviant stimulus. The three-way ANOVA showed a significant effect for the stimulus × lead [*F*_(1.945)_ = 13.937; *P* < 0.001] interaction and individual electrode sites were statistically analyzed. The significantly larger ERP amplitude in response to the deviant stimuli in the 90–130 latency range was shown for F3, Fz, F4, and M1 in the first block and F3, Fz, F4, C3, Cz, M1, and M2 in the second block of the oddball paradigm. The ERPs for the Fz are shown in the Figure 3.

Target stimulus elicited the N2b-P3 complex, which is typical for experiments that require responses to rare relevant stimuli. The three-way ANOVA of N2b revealed a stimulus × lead interaction [*F*_(1.997)_ = 4.704; *P* < 0.036] and the significant effect of P3 for lead [*F*_(1.966)_ = 4.628; *P* < 0.039]. *Post-hoc* analysis found no differences between standard and deviant stimuli in these intervals.

In the deviants-only paradigm, the target stimulus elicited N1, P2, and P3 components.

## Discussion

In the present study, participants performed absolutely identical motor tasks with the same maximal exertion in four conditions: the LT deviants-only, the LT oddball, the ST deviants-only, and the ST oddball paradigms. The necessity of discriminating between stimuli in the oddball paradigm led to the activation of the pre-attentive MMN mechanism, leading to the attention-switching to stimulus change (Näätänen et al., 2007, 2011). In our data, this fact was confirmed by the MMN—a special component of evoked potentials.

The progressive development of muscle fatigue observed throughout the experiment was confirmed by significant decrease in force production during the exercise, by reduction in the MVC and increase in the RPE after the blocks of work. We found a significantly lower rate of fatigue development (as evident by the compression force and MVC values) in the ST than in the LT deviants-only paradigm. The ST and the LT oddball paradigms took an intermediate position, differing significantly from the ST and the LT deviants-only paradigms, respectively. A positive effect of involuntary attention related to MMN generation was found for the LT: the force of contraction was greater when the motor task was performed in the oddball paradigm. The opposite effect was shown in the ST: the force of contraction was less in the oddball paradigm than in the ST deviants-only paradigm. Thus, in paradigms with identical motor tasks subjects decreased the work intensity in the longer condition, showing the significantly greater fatigue. The involuntary attention activation interfered with this process, resulting in the increased or decreased fatigue depending form the expected trial duration (shorter or longer, respectively).

The significantly lower contraction force in the long condition compared to the short, performed in the deviants-only paradigm, i.e., in control circumstances without involuntary attention activation, can be explained by the expected task duration influence, because these paradigms differed only by the prior knowledge about it. This idea is supported by previous studies that have shown that the subjective feeling of fatigue is influenced by task duration expectation: the participant describes higher levels of perceived exertion at the same time point during a shorter duration task compared to a longer duration task (Walster and Aronson, 1967; Rejeski and Ribisl, 1980; Baden et al., 2004; Lindsay, 2006). Further studies have shown the presence of subconscious regulation during the exercises of maximal capacity. Kay et al. (2001) found that when performing a 60 min self-paced cycling time trial interspersed by six “all out” sprints, participants were able to return power output to near initial values during the final maximal effort sprint. This suggests the existence of a subconsciously controlled motor unit recruitment reserve during the initial five sprints, that was recruited during the final sprint “end spurt” (St. Clair Gibson and Noakes, 2004). In the Halperin and colleagues' study (Halperin et al., 2014) the participants were instructed to produce maximal force with each contraction in the control, unknown, and deception conditions. It was found that when participants expected fewer numbers of contractions (deception condition) compared to the unknown number of contractions (unknown condition) they demonstrated greater MVCs. Under all conditions subjects applied greater forces in the last repetition relative to the previous one. Authors claimed that the participants were not applying true maximal forces and subconsciously suppressed them until the expectation of the final repetition. Therefore, we could assume that the expectation of exercise duration influenced the exercise regulation. This mechanism regulates the maximal contraction force so that the individual is able to complete the activity of different duration without bodily damage or premature fatigue.

The RPE values, in our study, were smaller in the first block of the LT but matched those of the ST in the second block. Therefore, despite stronger fatigue in the first block of the LT, RPE values were small. This finding is in agreement with the results reported by other authors (Rejeski and Ribisl, 1980; Swart et al., 2009), who found that exercise with an expected shorter duration causes greater RPE values. An inverse linear relationship between trial duration and rate of increase in RPE was shown by Crewe et al. (2008). Thus, expecting the greater trial durations subjects maintained the greater metabolic reserve, performing the task with less intensity and decreased RPE values. At the end of the exercise, but not before, the RPE matched the maximal tolerable value (Tucker, 2009). In our study, the rating of perceived exertion values for the oddball and deviants-only paradigms was similar at the end of the STs and LTs, indicating the presence of subconscious regulation of the exercise intensity.

Therefore, in our study the development of fatigue in the control (deviants-only) condition depends on the prior knowledge about the task duration. The activation of the involuntary attention interferes with this process resulting in increased fatigue in the shorter condition and decreased in the longer. The ability to influence on the maximal force value and the fatigue by different cognitive factors and the attention had previously been shown in some studies. Positive effect on recovery after fatigue was found when physical work and cognitive load were performed in the rest period (Setschenow, 1903; Asmussen and Mazin, 1978; Mathiassen et al., 2008). The modulation of repeated MVCs values during the exercise performance was reported by Ikai and Steinhaus (1961), who founded the occasional presence of the loud noise to increase the after-the-noise maximum-effort pulls. The positive influence of sustained vocal encouragement, motivational self-talk, motivational music and video on the power output during the maximal effort exercising (St. Clair Gibson and Noakes, 2004; Barwood et al., 2009, 2015) suggests the involvement of motivation and effort perception in regulation of maximal force output under different environmental conditions. Evstigneeva et al. (2012) reported that the button pressing in response to the rare deviant acoustic stimuli during the fatiguing task (sustained submaximal contraction) resulted in improved motor task performance, compared to the passive listening task.

Thus, the effect observed in the oddball paradigm may be associated with the interaction of motor and cognitive processes, related in this case to the discrimination between stimuli. Some groups reported the MMN activating effect (Aleksandrov et al., 2003, 2005): the reaction time and the number of errors in the acoustic signal recognition task were smaller when the MMN was invoked by the stimulus. Possibly, the MMN presence during the motor task performance in response to target stimuli enhanced the reaction related only to the perception of the stimulus, simultaneously affecting all other processes. In this case, the subconscious regulative mechanism can be suppressed and the task may be performed with the same intensity, regardless of the information about its duration, which results in the intermediate position of the force values obtained from oddball paradigms. Therefore, the activation of involuntary attention may cause different effects related to the expected task duration. The activation of the attention-switching mechanism leads to the destruction of the exercise regulative mechanism, and the force production increases in the long and decreases in the short tasks, compared to the control (deviants-only) condition.

In conclusion, we found that the central nervous system possibly regulates the fatigue development during the repeated contraction exercises of different duration. The MMN and the related involuntary attention activation interfere with this subconscious regulative mechanism. This process looks like increased fatigue during the ST and decreased fatigue during the LT in the oddball paradigm. This study may be applied as follows: the usage of sound sequences, resulting in attention activation (including music) has probable effect only in exercises of the expected prolonged duration, as it leads to a reduction of fatigue development. For short exercises, this approach seems ineffective.

## Author contributions

AA provided the concept of the study. AA and VK provided design of the study. VK, ED, and LS contributed data acquisition and analysis. AA, VK, LN, ED and AS contributed to the interpretation and discussion of the results, drafted, and critically revised the manuscript.

## Funding

The work of AA, VK, LS, and ED was supported by the Russian Science Foundation under Grant N_o_ 14-25-00065. The work of AS was supported by the subsidy granted to the HSE by the Government of the Russian Federation for the implementation of the Global Competitiveness Program.

### Conflict of interest statement

The authors declare that the research was conducted in the absence of any commercial or financial relationships that could be construed as a potential conflict of interest.

## Abbreviations

ANOVA: analysis of variance
ERP: event-related potential
FDR: false discovery rate
LT: long trial
MMN: mismatch negativity
MVC: maximum voluntary contraction
RPE: rating of perceived effort
SD: standard deviation
ST: short trial.
